# ASO Author Reflections: The Looming Challenge of Declining Reimbursements in Breast Cancer Care

**DOI:** 10.1245/s10434-024-15838-0

**Published:** 2024-07-31

**Authors:** Terry Gao, Kristen HoSang, Richard Bleicher, Lindsay Kuo, Austin Williams

**Affiliations:** 1 Temple University, Temple University Hospital, Philadelphia, PA USA; 2 Fox Chase Cancer Center, Philadelphia, PA USA

## Past: Key Clinical Question

Breast cancer remains a leading public health concern and Medicare serves as a vital source of financial support for many patients undergoing surgery. While past studies documented declining reimbursement rates across various surgical specialties, a comprehensive analysis dedicated to breast surgery was lacking. This lack of data is particularly concerning given ongoing changes in government policies and reimbursement legislation. For example, recent debates surrounding the Medicare Physician Fee Schedule conversion factor highlight the complex interplay between reducing administrative burden for providers and ensuring fair compensation.^1^ In 2021, proposed policy changes not only aimed to streamline documentation but also threatened significant cuts to overall physician payments. While Congress intervened to prevent immediate cuts, this episode exemplifies the ongoing struggle to balance budgetary concerns with adequate reimbursement for essential services.^2^ These ongoing debates over reimbursement legislation have likely led to impacts on breast surgery reimbursement, making it even more crucial to understand current trends.

Our study investigating trends in Medicare reimbursements for 10 common breast cancer operations over two decades (2003–2023), sheds light on a critical issue with significant implications for the future of breast cancer care.^3^

## Present: Relevance and Importance

While unadjusted data showed an increase in reimbursement rates, after accounting for inflation, we observed a concerning decline (20.70%) across most procedures. This misalignment between rising costs and stagnant reimbursements threatens the financial sustainability of breast cancer surgery practices. Our estimate suggests that in 2023, surgeons could face a shortfall of nearly $110 million compared with if rates had kept pace with inflation.

These results hold significant importance for several reasons. First, they highlight a trend that could potentially strain the financial sustainability of breast cancer surgery practices. Second, declining reimbursements threaten access to essential care for Medicare beneficiaries, a population particularly vulnerable due to their reliance on this program. Furthermore, this trend aligns with observations in other surgical specialties, suggesting a broader systemic issue within healthcare financing. Our study provides valuable data to support advocacy efforts aimed at ensuring fair compensation for breast cancer surgery procedures within the Medicare system.

## Future: Addressing the Challenge

Our study underscores the need for a multifaceted approach to address the challenge of declining reimbursements. Surgeon participation in the Medicare RVU review process, collaboration with professional societies for evidence-based advocacy, and partnering with hospital administrators for fair reimbursement structures are all essential steps.^4^ Policy advocacy efforts informed by our research findings can be instrumental in influencing healthcare policy changes.^5^ Additionally, exploring collaborations with other breast cancer care providers holds promise for developing cost-effective pathways that improve overall value delivery.

Beyond the limitations acknowledged in the manuscript, future research should encompass the broader healthcare ecosystem, including the impact on private insurers and provider financial sustainability through investigations into overhead costs and profit margins. Most importantly, future research must delve into how declining reimbursements affect patient outcomes, particularly regarding access to specialists, treatment decisions, and long-term well-being. This knowledge is paramount for informing future policy changes and ensuring equitable access to high-quality breast cancer care for all.
